# Effects of larval crowding on the transcriptome of *Drosophila simulans*

**DOI:** 10.1111/eva.13592

**Published:** 2023-09-27

**Authors:** Stephan Buchner, Sheng‐Kai Hsu, Viola Nolte, Kathrin A. Otte, Christian Schlötterer

**Affiliations:** ^1^ Institut für Populationsgenetik Vetmeduni Vienna Vienna Austria; ^2^ Vienna Graduate School of Population Genetics Vetmeduni Vienna Vienna Austria; ^3^ Present address: Institute for Zoology University of Cologne Cologne Germany

**Keywords:** *Drosophila simulans*, larval crowding, phenotypic plasticity, transcriptomics

## Abstract

Larval crowding is one common ecological stressor for many insect species. In *Drosophila*, high larval density alters multiple widely‐studied phenotypes including life‐history traits, morphology and behavior. Nevertheless, we still miss a holistic view of the full range of phenotypic changes and the underlying molecular mechanisms. In this study, we analyzed the adult transcriptomes of high and low larval density fly cohorts, and highlighted the molecular basis of the plastic traits. Increased cellular energy metabolism and locomotion, along with reduced reproductive investment, are key responses to high larval density. Moreover, we compared the expression changes among cohorts with different developmental delays caused by larval crowding. The majority of genes induced by larval crowding showed the strongest expression alterations in cohorts with intermediate delay. Furthermore, linear expression changes were observed in genes related to nutrition and detoxification. Comparing different high‐density cohorts could provide insights into the varied responses to distinct larval crowding‐induced stresses such as space competition, food degradation and waste accumulation.

## 1 INTRODUCTION

Environmental conditions during development have a significant impact on adult phenotypes and fitness (e.g. (Dufty et al., 2002)). The high population densities during juvenile development observed in nature (e.g. Atkinson, 1979) have triggered continued interest in plastic phenotypic responses to this environmental stressor (Pearl & Parker, 1922). For many insect species, larval or juvenile density has a noticeable impact on various aspects of development, reproduction, and survival. Higher densities generally lead to longer development times, changes in body size and proportions, increased sperm production, decreased adult weight, and sometimes reduced survival rates (Credland et al., 1986; Gage, 1995; Lyimo et al., 1992; Stockley & Seal, 2001). Similarly, in *Drosophila*, high rearing density during larval development causes a broad spectrum of phenotypic changes. Among the best documented phenotypes are decreased larvae‐to‐adult viability (Blondel et al., 2016), increased heterogeneity in developmental rate (Lints & Lints, 1971; Lushchak et al., 2019), reduced body size (Klepsatel et al., 2014), altered morphology (Imasheva & Bubliy, 2003), decreased fecundity (Lints & Lints, 1971), increased stress resistance (Henry et al., 2018; Lushchak et al., 2019), altered adult behaviors (Ribó et al., 1989), and increased longevity (Klepsatel et al., 2018). Many of these phenotypic changes were observed in adult flies, highlighting the influence of developmental conditions on adult phenotypes and even fitness (Morimoto et al., 2017; Poças et al., 2022). Similar impact on adult traits of high larval density was also documented in other insects (Credland et al., 1986; Gage, 1995; Lyimo et al., 1992; Stockley & Seal, 2001). While the impact of high larval density on stress‐related phenotypes has been extensively studied, there is still a need for a comprehensive survey that investigates the range of affected phenotypes, specifically focusing on the associated molecular mechanisms.

The phenotypic plasticity in response to larval crowding can be triggered by many but not mutually exclusive stressors including the increased physical interaction due to spatial constraint, limited resource availability due to competition (Klepsatel et al., 2018), and increased concentration of waste products (Botella et al., 1985; Henry et al., 2018). Some studies focused on the impact of dietary limitation on several life‐history traits (Klepsatel et al., 2018) while others demonstrated a connection between the delayed developmental rates and the uric acid and urea content in the food (Botella et al., 1985). Similarly, the hormesis‐like effect induced by mild larval crowding was also linked to the accumulation of toxic waste (Henry et al., 2018). While the influence of certain stressors resulting from larval crowding is documented for specific phenotypes, our current understanding lacks a holistic view of the full range of phenotypic changes. In this study, we propose that leveraging the observed variation in developmental rates induced by high larval densities may provide insights to the existing knowledge gap.

The time of eclosion can be associated with phenotypic differences of adult flies because larvae experience different micro‐environments across time. For instance, resources become more limited and waste products accumulate with time, which may lead to altered phenotypes of adults eclosing at different time points. On the other hand, physical interactions between larvae due to spatial limitation may change in a more complex manner. Rapidly developing larvae will experience less spatial limitation. Similarly, the slowest developing larvae may suffer less limitation in space because a large fraction of larvae has already pupated and no longer compete with them. The largest competition for space is expected for larvae with intermediate developmental rates. We propose that a systematic comparison between the fly cohorts with different developmental rates will shed light on distinct stressors induced by high larval density and potentially elucidate their impacts on adult physiology.

Gene expression profiling is a well‐established method to obtain a large number of molecular phenotypes (i.e., gene expression) across a wide range of biological processes. We used RNA‐Seq to identify the phenotypic changes of adult flies, which were kept at the same density at adult stage, but were exposed to different larval densities during their development. Our analyses show that the gene expression of 22.5% of the expressed genes is significantly altered, covering broad functional classes. The comparisons among fly cohorts exhibiting differential development rates in high rearing density may help distinguish between life‐history shifts influenced by space competition, stress responses due to environmental degradation, and potentially other unknown factors.

## 2 MATERIALS AND METHODS

### 2.1 Culturing conditions for phenotyping

**FIGURE 1:**
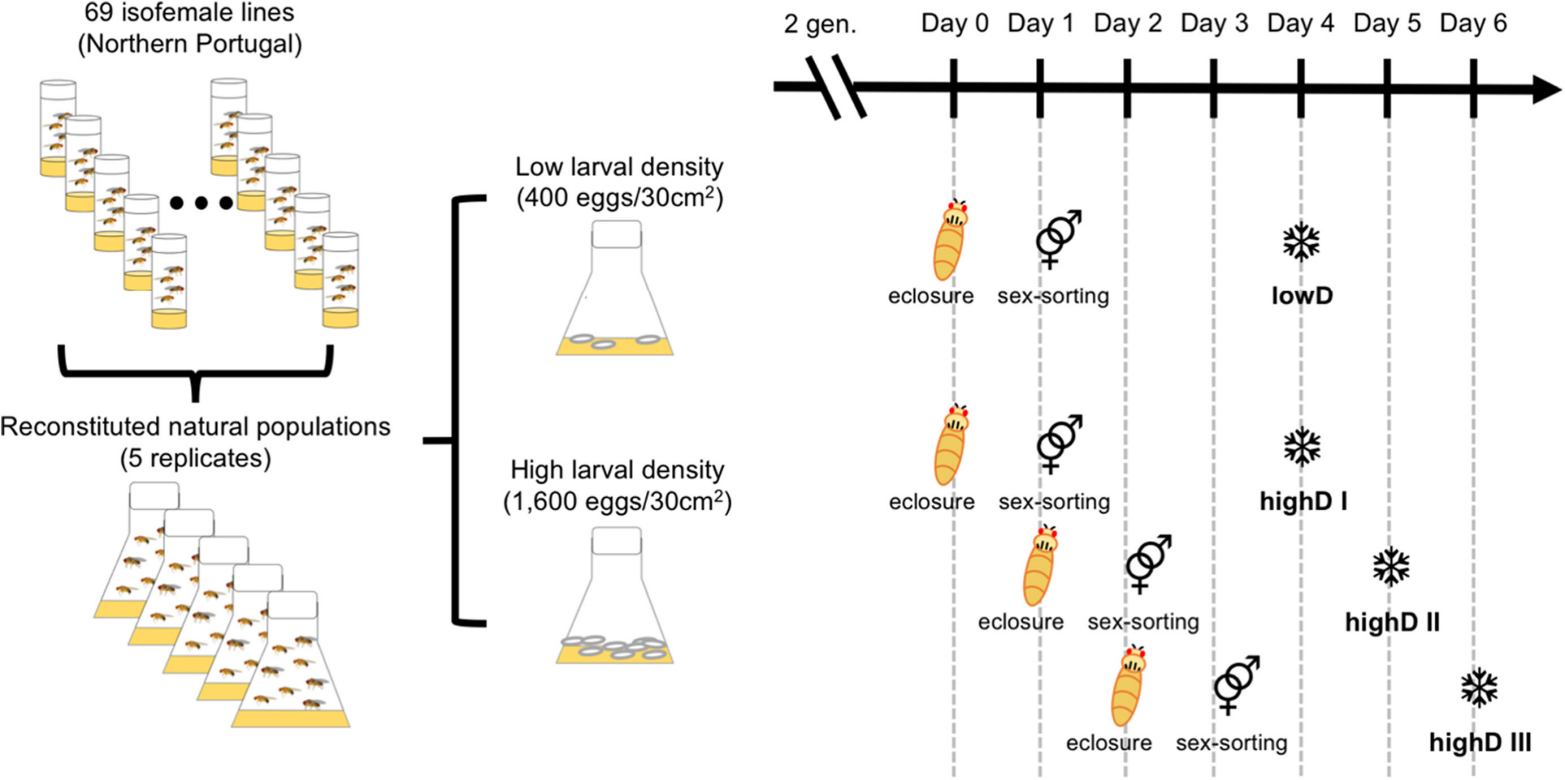
Experimental procedure. 69 isofemale lines collected from Portugal were pooled in equal proportions to reconstitute five replicates of the natural population. The flies were reared at 28/18°C with 12 h‐12 h light/dark cycle for two generations at low density and high density before being subjected to collection and sequencing. At the low‐density condition, all flies eclosed on the 11th day after egg lay. Eclosed flies were allowed to mate randomly for 1 day. Afterwards, 50 males were collected into a 30 mL‐vial for recovery from anesthesia for 2 days. On the fourth day after eclosure, the flies were snap frozen in liquid nitrogen and stored at −80°C. High larval density results in heterogeneous developmental rates. Thus, three cohorts of flies were collected in 24‐h intervals. The same collecting protocol applies to all cohorts. All flies were frozen at the age of 4 days.

A *Drosophila simulans* population collected 2008 in Northern Portugal was maintained until the start of the experiment as 69 isofemale lines. These 69 isofemale lines were pooled in equal proportions to reconstitute the natural population in five replicates. The flies were reared at 28/18°C with 12 h‐12 h light/dark cycle for two generations at low density and high density before being subjected to collection and sequencing (Figure 1). For the low rearing density treatment, 400 eggs were put into the rearing bottles with a surface area of 30 cm^2^ (90 mL) using a pipetting method (Nouhaud et al., 2018) while four times as many eggs (1600) were used for the high rearing density.

At the low‐density condition all flies eclose on the 11th day after egg lay. Eclosion of flies within a single day at low‐density conditions has been described before (Lushchak et al., 2019). High larval density results in heterogeneous developmental rates with flies eclosing over a longer period ranging from 11 to 13 days after egg lay. Reasoning that flies eclosing at different days were experiencing different environments, we collected high‐density flies in 24 h intervals (Figure 1). Thus, four cohorts of flies were collected (low rearing density on the 11th day after egg lay (lowD), high rearing density samples collected on the 11th day (highD I), high rearing density samples collected on the 12th day (highD II) and high rearing density samples collected on the 13th day (highD III)). Thus, the developmental time of low‐density flies and the first cohort of high‐density flies was identical, as described previously (Lushchak et al., 2019). For each cohort, five biological samples were collected and the two sexes separated under light CO_2_ anesthesia. Each sample comprised 50 males snap‐frozen using liquid nitrogen and stored at −80°C at the age of 4 days after eclosion. Each replicate contained the same number of flies.

### 2.2 RNA‐Seq library preparation

After removal from the −80°C storage flies were immediately immersed and homogenized in Qiazol (Qiagen). Total RNA was extracted from whole body males using the Qiagen RNeasy Universal Plus Mini kit. RNA‐Seq libraries were prepared with the TruSeq stranded mRNA Library Prep Kit on a Neoprep device (software version 1.1.0.8 and protocol version 1.1.7.6, Illumina) starting with 100 ng of total RNA and using the default settings with an insert size of 200 bp and 15 PCR cycles. We avoided batch effects by randomizing all libraries across library cards with an identical lot number. 50 bp reads were sequenced on the Illumina HiSeq 2500 platform.

### 2.3 RNA‐Seq data processing

Sequencing quality was assessed with FastQC (Wingett & Andrews, 2018). Reads were trimmed using a modified Mott algorithm, implemented by the TrimReads function of the package “readtools” (Gómez‐Sánchez & Schlötterer, 2018) with a minimal quality score of 20. The trimmed reads were mapped to a *D. simulans* reference genome (Palmieri et al., 2015) with GSNAP (Wu et al., 2016) using the parameters ‐m 0.08 ‐N 1, which allow for a maximum of eight percent mismatch and the identification of novel splicing events. Exon‐aligned reads were counted with Rsubread (Liao et al., 2019). Library sizes and read alignment statistics are given in Table S1. We determined gene body coverage with RSeQC (Wang et al., 2012) to identify libraries with quality problems, probably caused by the Neoprep library preparation. One sample of the highD III cohort was excluded (Figure S1). Re‐running this sample was not possible because no library cards of the same lot were available anymore. The expression values were normalized based on TMM algorithm implemented in edgeR (Robinson et al., 2010). Only genes with more than one count per million in all samples were considered for subsequent analysis.

### 2.4 Differential expression analysis

Principal component analysis (PCA) of the log transformed CPM of all expressed genes was used for the comparison of global expression patterns among all samples.

Genes affected by rearing density were identified based on likelihood ratio test framework implemented in edgeR (Robinson et al., 2010). A contrast was made for all highD cohorts against the lowD cohort. We accounted for multiple testing using the Benjamini‐Hochberg procedure (Benjamini & Hochberg, 1995). Subsequent analyses were done for genes with significant differences in gene expression (FDR < 0.05) and at least 1.25‐fold change (FC).

We used Kruskal–Wallis test (R function *kruskal* () in *agricolae* package) to test for expression differences among all possible pairwise combination of cohorts for the genes significantly affected by rearing density. Post hoc grouping is used to determine the statistical difference of the expression levels among different cohorts.

### 2.5 Weighted gene co‐expression network analysis

Genes with a co‐regulated gene expression pattern across samples were identified with a weighted gene co‐expression network analysis (WGCNA) (Langfelder & Horvath, 2008). A signed network was constructed with a minimal module size of 100 genes and the argument reassign Threshold of 10^−4^, for all other parameters we used standard settings.

### 2.6 Functional enrichment analysis (GO analysis)

Gene ontology (GO‐terms) enrichment was conducted with the R package “TopGO” (Alexa et al., 2006) and the “weight01” algorithm. Only terms with more than five significantly differentiated genes were considered. The GO annotation and gene ID mapping were based on the database for *Drosophila melanogaster* (namely, R objects “annFUN.org” and “org.DM.eg.db” in R/BioConductor V3.10).

## 3 RESULTS

### 3.1 Transcriptomic plasticity in high rearing density

**FIGURE 2:**
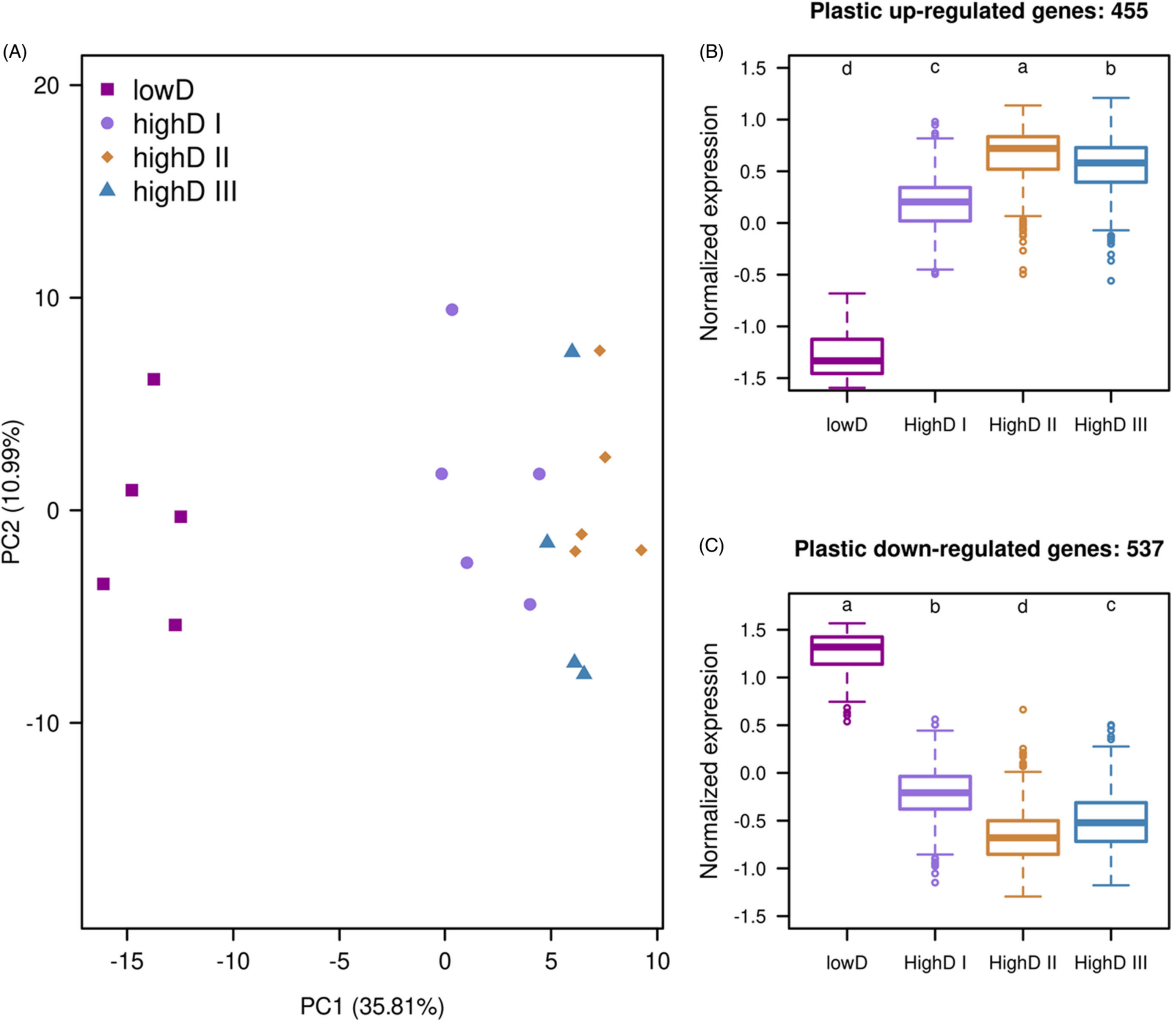
Transcriptomic divergence in response to high rearing density. (A) Principal component analysis (PCA) of the expression values of all 10,821 expressed genes. ~36% of the transcriptomic variation is explained by the first principal component (PC1), which separates flies reared in low density (lowD, purple square) and high density (in other colors and shapes). On the same axis, fly cohorts with differential delay in development were also separated (highD I: light purple circle, highD II: yellow diamond, highD III: blue triangle). (B and C) The normalized expression of plastic genes in response to high rearing density for up‐ and down‐regulation is plotted. The colors indicate different cohorts. Post hoc significance tests show that all cohorts differ significantly from each other as indicated by four different letters (a–d) (*p*‐values of all post‐hoc comparisons <2.2 × 10^−16^ except for the difference between highD II and highD III for the up‐regulated genes where the post‐hoc *p*‐value = 1.9 × 10^−12^). Thus, the genes are not only expressed differentially between the LD and each of the HD cohorts, but also among the three HD cohorts.

A principal component analysis (PCA) on the global transcriptomic variation of 10,781 expressed genes clearly differentiated cohorts reared at low and high density on the first principal component (PC1), which explained 35.81% of variance in the data set (Figure 2A). Among the high‐density cohorts, PC1 also distinguished cohorts with different developmental rates. Flies from high‐density treatment which eclosed at the same time after egg laying (highD I) were the least differentiated from the flies from low density (lowD). Interestingly, the flies eclosed the latest (two‐day delay, highD III) were not the most differentiated ones, but grouped between cohort highD I and highD II (Figure 2A).

We identified genes with significant gene expression change in response to high rearing density by contrasting samples reared in low density to all samples reared in high density. Out of 10,781 genes expressed in all populations, 2425 (22.5%) were significantly differentially regulated at high rearing density (Table S1). Among them, 992 genes exhibited more than 1.25‐fold expression difference between the high‐ and low‐density treatments (Table S2). 455 of these plastic genes were up‐regulated and 537 were down‐regulated.

We linked density‐related transcriptomic plasticity to the potential underlying biological processes and organismal traits using GO enrichment analysis for the genes with significantly different gene expression at high rearing density. Up‐regulated genes in high‐density conditions are involved in energy production (e.g. “mitochondrial electron transport, NADH to ubiquinone” (GO: 0006120), “mitochondrial electron transport, ubiquinol to cytochrome” (GO: 0006122) and “ATP synthesis coupled proton transport” (GO: 0015986)) and muscle activity (e.g. “skeletal myofibril assembly, regulation of muscle contraction” (GO: 0006937), and “musculoskeletal movement” (GO: 0050881)) (Table S3). On the other hand, genes exhibiting lower expression in high density are enriched for reproductive functions (e.g. “multicellular organism reproduction” (GO: 0032504) and “sperm storage” (GO: 0046693)), immune responses (e.g. “response to bacteria” (GO: 0009617), “response to fungus” (GO: 0009620) and “humoral immune response” (GO: 0006959)), and some metabolic processes (e.g. “tyrosine metabolic process” (GO: 0006570), “gluconeogenesis” (GO: 0006094), and “cellular lipid metabolic process” (GO: 0044255)) (Table S3). Repeating the GO enrichment analysis on genes showing at least two‐fold expression differences (67 and 46 genes for up‐ and down‐regulation respectively) re‐capitulated the importance of increased muscle activity and reduced immune responses/metabolic budgets in adult flies experienced high larval density (Table S4).

### 3.2 Differential plasticity among high‐density cohorts

Although all three high‐density cohorts were clearly distinct from the low‐density cohort, expression differences were observed between them (Kruskal‐Wallis test, Figure 2). We propose that changes in micro‐environmental conditions (i.e. food quality, waste concentration, space competition etc.) may have occurred, which in turn triggered the heterogeneous expression response among high‐density cohorts with differential developmental rates. We performed a weighted co‐expression network analysis (WGCNA) with all four cohorts to nominate potential stress components based on characteristic expression profiles of the underlying genes.

#### 3.2.1 Most high‐density‐induced genes exhibit the strongest responses in highD II cohorts

**FIGURE 3:**
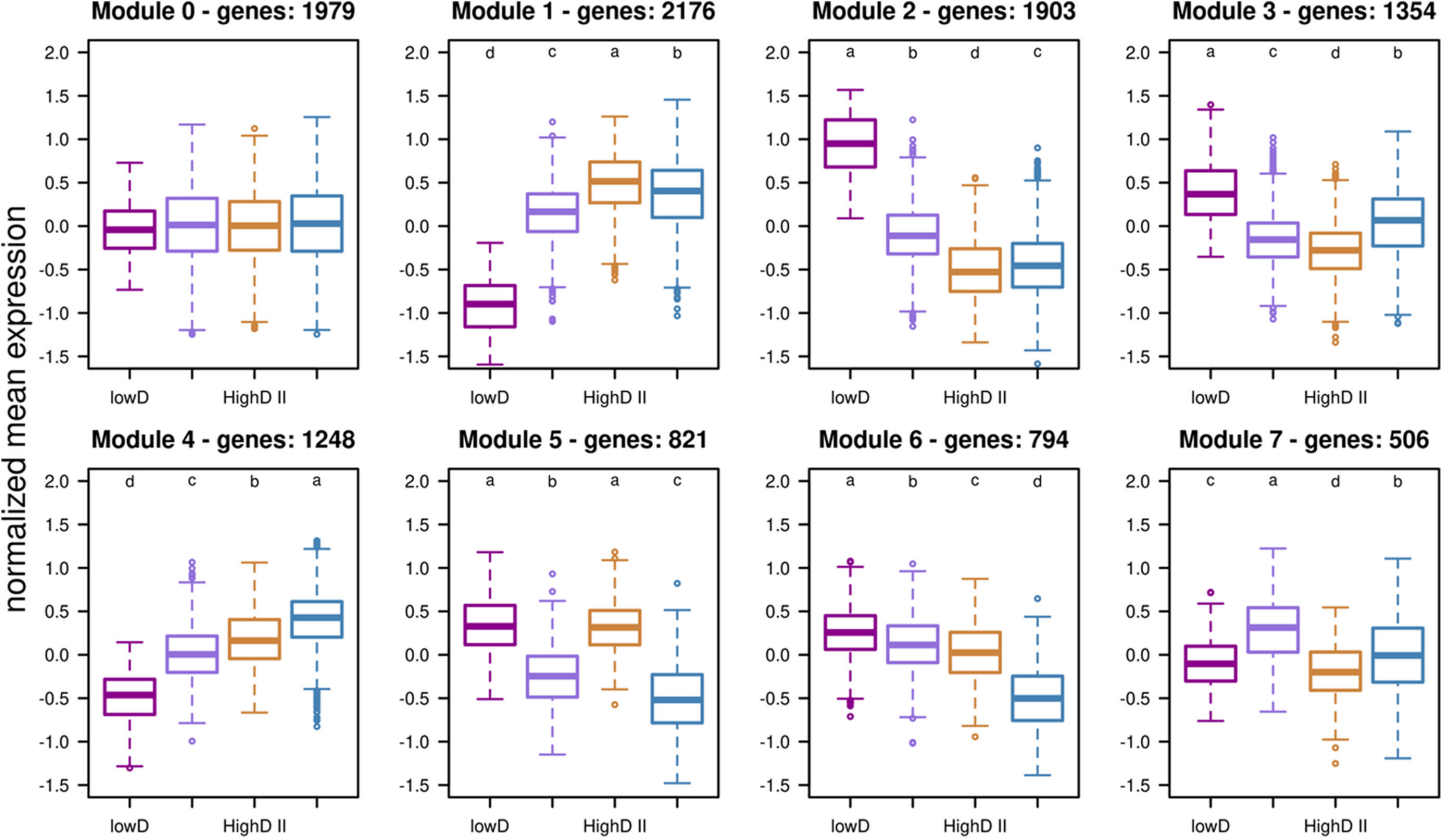
The expression pattern of co‐expressed modules. The normalized expression of plastic genes classified in different modules is plotted. The colors indicate different cohorts. Module 1, 2 and 3 may share a stress factor that is the strongest for the high‐density cohort with intermediate developmental rate. Module 4 and 6 captures genes which respond to a stressor that increases with developmental time among the high‐density cohorts. Most post‐hoc comparison among groups are statistically significant (Module 2: *p*‐value = 1.6 × 10^−7^ for the difference between highD II and highD III; Module 5: *p*‐value = 0.23 for the difference between lowD and highD II; Module 6: *p*‐value = 7.0 × 10^−8^ for the difference between lowD and highD I; Module 7: *p*‐value = 6.2 × 10^−7^ for the difference between lowD and highD 5.0 × 10^−4^ between lowD and highD II and 8.6 × 10^−13^ between highD II and highD III; all other *p*‐values <2.2 × 10^−16^).

We identified eight co‐regulated modules with distinct expression patterns among the four cohorts (Figure 3). Module 1 and Module 2, which consist of more than 1000 genes each, were enriched for significantly up‐ and down‐regulated plastic genes (Fisher's exact test, *p*‐value <2.2e‐16 in both tests), as defined by the contrast of high‐ and low‐density flies. More than 90% of the plastic genes were assigned to these two modules. Interestingly, developmental rate and plasticity intensity were not correlated among the high‐density cohorts (Figure 2B,C; Figure 3; Module 1 and 2). Rather, highD II cohort exhibited the largest transcriptomic response relative to the lowD cohort. Module 3 (1354 genes) provides further support for the special position of the highD II cohort, because it displayed the largest differences in gene expression (Figure 3).

Given the large overlap with high‐density‐induced plastic genes, similar GO terms were enriched for Module 1 and 2 (Table S4). Up‐regulation of energy metabolism and muscle activity as well as the down‐regulation of reproduction and immune response are the most prominent functional enrichments. Module 3 contains a distinct set of genes involved in the development of nervous system (e.g. “axon guidance” (GO: 0007411), “dendrite morphogenesis” (GO: 0048813) and “ventral cord development” (GO: 0007419)), memory formation (e.g. “long‐term memory” (GO: 0007616), and “mushroom body development” (GO: 0016319)). Similar to module 2, reproduction‐related genes were also down‐regulated in module 3 (e.g. “sperm individualization” (GO: 0007291), and “gonad development” (GO: 0008406)).

#### 3.2.2 Linear expression changes associated with developmental delay at high rearing density

In addition, we identified two co‐expressed modules containing fewer but not a non negligible number of genes with expression continuously increasing/decreasing with developmental time at high rearing density (Figure 3: Module 4 and 6). We propose that these two modules may reflect the distinct axis of density‐induced stress that increases over time.

We performed GO enrichment analysis for these two modules (4 and 6) in order to identify the altered biological processes. Genes in module 4 were enriched for GO categories including processes responding to waste products and limitation of nutrition (e.g. “behavior response to nutrients” (GO: 0051780), “response to salt stress” (GO: 0009651), “response to starvation” (GO: 0042594), and “cellular response to decreased oxygen levels” (GO: 0036294)). Furthermore, genes were also enriched for GO categories related to behavioral control (e.g. “larval locomotory behavior” (GO: 0008345), “male courtship behavior” (GO: 0008049), “social behavior” (GO: 0035176), and “chemosensory behavior” (GO: 0007635)). On the other hand, genes with stress causing lower expression (Module 6) were related to proteolysis e.g. “proteasome‐mediated ubiquitin‐dependent protein catabolic process” (GO: 0043161), several metabolic processes e.g. “malate metabolic process” (GO: 0006108), and the transport e.g. “malate transmembrane transport” (GO: 0071423).

## 4 DISCUSSION

Until now, high larval density was studied for a broad range of phenotypes, mostly related to life history (Blondel et al., 2016; Klepsatel et al., 2018; Lints & Lints, 1971). This study did not focus on a set of predefined high‐level phenotypes, but rather used gene expression to explore a wider range of molecular phenotypes. We aimed for better understanding of the molecular basis and thorough exploration of phenotypic plasticity induced by high larval density. The objective is in line with a recent study in *D. melanogaster* (Morimoto, 2022) where transcriptomic comparisons were made in larvae reared in high, medium and low density. We performed comparative transcriptomic analysis on adult cohorts reared in high and low larval density and observed pronounced gene expression plasticity with about 22.5% of all expressed genes being significantly different between the two treatments. Interestingly, a significant proportion of the high‐density‐induced genes identified in this study also exhibited differential expression in the same direction as observed in a recent comparative transcriptomic study conducted at the third‐instar larval stage (Morimoto, 2022) (Fisher's exact test; odds ratio = 3.78 and 2.54; *p*‐value <2.2 × 10^−16^ and *p*‐value = 1.8 × 10^−14^ for the up‐ and down‐regulated genes respectively). Consistent with previous phenotyping observations (Morimoto et al., 2017; Poças et al., 2022), this result highlights that stress responses during larval stage are carried over to the adult stage, although adults were kept at the same density. The genes with significant changes in expression were related to metabolism, cellular energy production, the immune system, reproduction, and locomotor behavior. These findings are concordant with previous phenotypic observations and measurements in life‐history trade‐off and/or behavior (Blondel et al., 2016; Henry et al., 2018; Imasheva & Bubliy, 2003; Klepsatel et al., 2014, 2018; Lints & Lints, 1971; Lushchak et al., 2019; Ribó et al., 1989). The identification of the differentially regulated genes and modules augmented our understanding of the molecular basis of the phenotypic plasticity.

The majority of previous experiments has focused on either the differences between high and low densities (e.g. (Lints & Lints, 1971)) or across a gradient of different densities (e.g. (Henry et al., 2020)). Although the heterogeneous developmental rate at high density is well documented (Lints & Lints, 1971), this heterogeneity within high‐density treatments was frequently not included in the data analysis. Unprecedentedly, we compared the magnitude of changes in high‐density‐induced phenotypes and documented interesting patterns of gene expression plasticity in high‐density cohorts with different developmental rates.

We identified in a weighted co‐expression network analysis (WGCNA) two modules (4 and 6) of genes for which expression increased or decreased continuously with developmental time in high rearing density. Most of the up‐regulated genes are relevant to the responses to starvation and salt stresses. These include several functionally characterized genes: *Carnation* (*Car*; *FBgn0000257*) and *Autophagy‐related 1* (*Atg1*; *FBgn0260945*) were shown to take part in starvation‐induced autophagy (Melani et al., 2017; Takáts et al., 2014). *Aps* (*FBgn0036111*) plays an important role for starvation resistance (Williams et al., 2015). *NFAT* (*FBgn0030505*) transcription factor is a key regulator promoting salt tolerance. One potential factor contributing the linear relationship between stress responses and delayed in developmental rate is the continuous decay of food media with high larval density. Nutrient limitation and waste accumulation would be more severe for slowly developing cohorts. A similar continuous change was previously seen in males eclosed at high‐density conditions for high‐level phenotypes, such as weight and maximum life span (Lushchak et al., 2019). The authors attributed these phenotypic changes to hormesis‐like effects, which increased the stress response. The continuous increase of gene expression for genes related to behavior is also interesting as it suggests that the social behavior of adult flies may differ within cohorts of different developmental times after experiencing high larval density. We anticipate that this may trigger a series of follow‐up experiments focusing on this specific aspect.

It is, nevertheless, apparent that the majority of high‐density‐induced genes followed a distinct expression pattern. Most of the high‐density‐induced genes showed the strongest responses in highD II cohort (Module 1 and 2). The GO enrichment analysis of these genes suggest a shift in energy allocation of adult flies reared in high larval density: increased energy production and muscle activity but declined reproduction. This provides a potential mechanistic explanation to the observed shift in life‐history trade‐off in high‐density condition (Blondel et al., 2016; Klepsatel et al., 2018; Lints & Lints, 1971). The most pronounced transcriptomic modification in highD II cohort is re‐iterated by the identification of co‐expression module 3 and 12 where genes are differentially expressed in highD II cohort than all other cohorts. Module 3 is particularly interesting as it suggests that the cohorts highD II and, to a lesser extent, highD I experience different neuronal development, which affects for example long‐term memory. Further studies are needed to understand the behavioral differences of flies from different high‐density cohorts and their evolutionary implications. We propose that a stressor triggered by space limitation, such as the increased larval physical interactions, may be the key determinant for the observed differences in gene expression. The extreme pattern of the highD II cohort may be attributed to the highest larval density for this cohort, because for highD I, the delayed development of the other cohorts results in less pronounced space limitation, and for highD III, this stress is already relaxed as the actual density before pupation for this cohort might be lower when all other flies have pupated already.

Based on these results, we suggest that distinct stressors induced by high larval density may impact the fly cohorts with distinct developmental rates in high larval density in different ways. However, it is important to keep in mind that we exploited the heterogeneity in developmental rate at high‐density conditions in the analysis. We reasoned that the identified genes with a certain gene expression pattern reflect the environment where each cohort experienced. Nevertheless, the expression difference among highD cohorts could as well be the indirect result of different developmental rates per se. More likely, the expression patterns observed here come from a combinatory effect of both factors. Furthermore, the high heterogeneity in developmental time has also been observed for inbred lines (Lints & Lints, 1971; Lushchak et al., 2019), which rules out genetic differences between the cohorts with different developmental times. In this study, we used a polymorphic population which was exposed to different stress levels. Hence, we cannot rule out that the differences in developmental time reflect genetic differences between adult males collected at different time points. We anticipate that the presented results will spearhead novel experiments to tackle the interaction between micro‐environment, development, and genetics.

Moreover, it is also important to understand the fitness consequence of the documented phenotypic plasticity in response to larval crowding as it is a common stressor for insect population in nature (Pearl & Parker, 1922). Particularly, whether or not the carried‐over phenotypic changes in adults observed in this study contributes as adaptive strategies for the next generation is an interesting question. A recent experimental evolution study in *D. melanogaster* showed that more than 100 generations of adaptation to larval crowding resulted in a size reduction of male reproduction organs, but this plastic response to larval crowding treatment did not change (Kapila et al., 2022). This suggests that the size reduction of adult reproductive organs in response to larval crowding may not provide a fitness advantage. Further exploration on other phenotypes and/or the full transcriptome will provide us a more complete pictures on the fitness consequence of the larval crowding‐induced phenotypic changes in adults.

Last but not least, many of the documented plasticity‐related genes in *Drosophila* may also be affected in other animal species when experiencing larval/juvenile crowding. Juvenile crowding occurs frequently as a common stressor for many species in nature (Pearl & Parker, 1922). Although not as extensively documented as in the model organism *Drosophila*, phenotypic plasticity related to high growing density has been studied in several other animal species, stressing its general importance in ecological studies. Similar shifts in life‐history traits in response to larval density were documented in multiple insect species (Credland et al., 1986; Gage, 1995; Lyimo et al., 1992; Stockley & Seal, 2001). Dramatic behavior and morphological changes in response to early stage crowding have been reported in desert locusts (Pener & Simpson, 2009; Simpson et al., 2001). Not only in insects, population crowding is also relevant to reproduction and behavior in higher animals such as fishes (Fenderson & Carpenter, 1971). Hence, we anticipate this study in *Drosophila* would serve as a model resource for the community for future studies in other important species, specifically on pinpointing the potential molecular basis of plastic phenotypes.

## CONFLICT OF INTEREST STATEMENT

The authors declare no conflict of interest.

## Supporting information


Figure S1.

Table S1.

Table S2.

Table S3.

Table S4.

## Data Availability

Raw sequence reads generated in this study are available in European nucleotide archive (ENA) under the study accession number of PRJEB48311. Tables [Link], [Link], intermediate files and associated R scripts are available on Github repository of this study (https://github.com/ShengKaiHsu/density_plasticity_Dsim_SA).
