# Supplementary material for: Effects of larval crowding on the transcriptome of Drosophila simulans
Source: Evol Appl. 2023 Sep 27;16(10):1671–9. doi: 10.1111/eva.13592 (PMC10660784; doi:10.1111/eva.13592)
Supplement: Supplementary file 1 — Figure S1. [file EVA-16-1671-s004.docx]

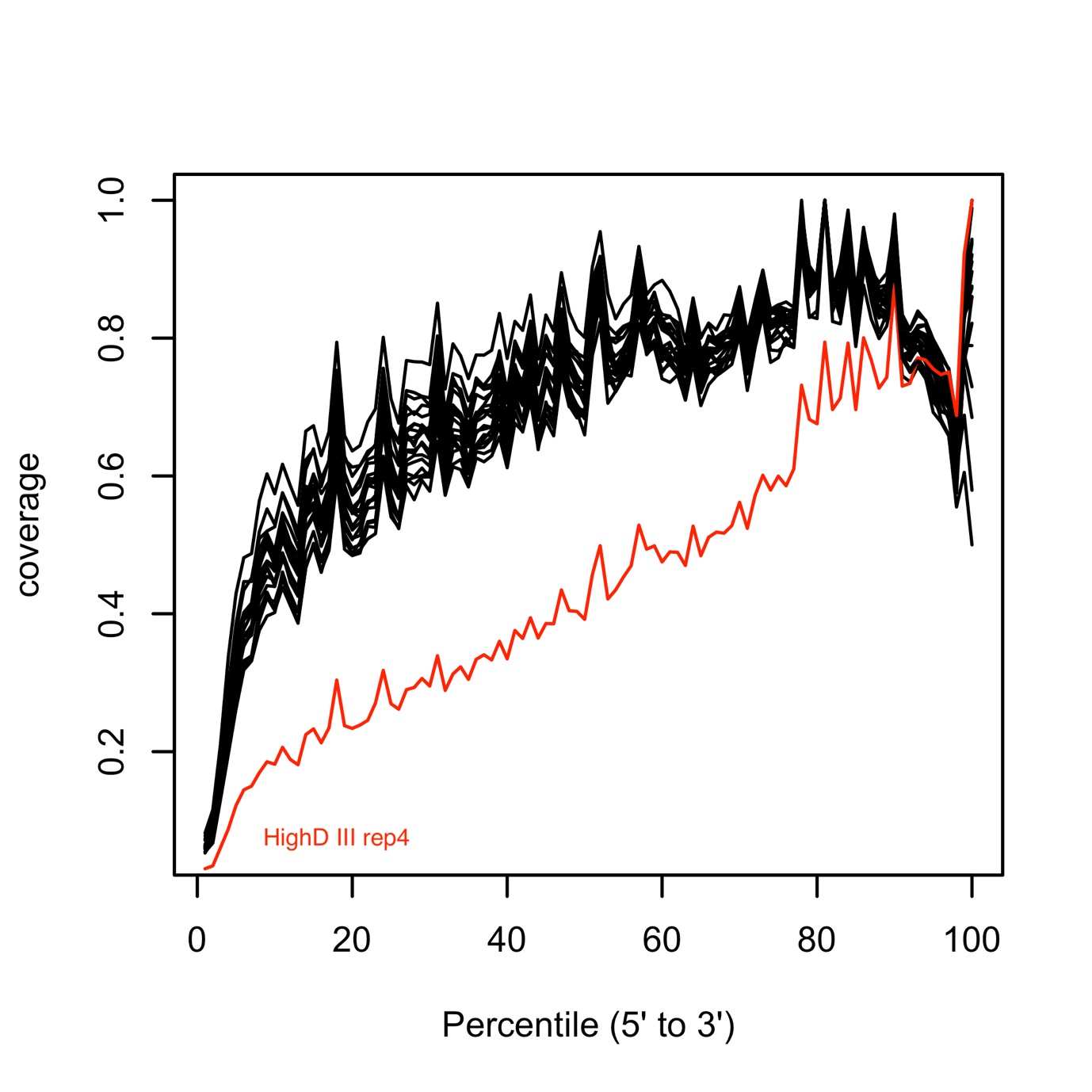


**Figure S1. Quality check for 3’-bias of RNASeq libraries.** To assess the integrity of mRNA molecules within the libraries, we examined the distribution of read coverage along the transcripts. Libraries containing degraded mRNA tend to exhibit a biased coverage pattern towards the 3'-end of the molecule. In our analysis, we identified one sample from the highD III cohort that displayed such bias and, as a result, was excluded from subsequent analysis.
